# A new Terrarana frog of genus *Pristimantis* from an unexplored cloud forest from the eastern Andes, Colombia

**DOI:** 10.3897/zookeys.961.51971

**Published:** 2020-08-19

**Authors:** Andrés R. Acosta-Galvis, Ana M. Saldarriaga-Gómez, Beatriz Ramírez, Mario Vargas-Ramírez

**Affiliations:** 1 Colecciones Biológicas, Subdirección de Investigaciones, Instituto de Investigación de Recursos Biológicos Alexander von Humboldt, Carrera 8 No 15–08, Claustro de San Agustín, Villa de Leyva, Boyacá, Colombia Instituto de Investigación de Recursos Biológicos Alexander von Humboldt Boyacá Colombia; 2 Grupo Biodiversidad y Conservación Genética, Instituto de Genética, Universidad Nacional de Colombia, Bogotá, Colombia. Calle 53 # 35–83, Edificio 426, Bogotá D.C., Colombia Universidad Nacional de Colombia Bogotá Colombia; 3 Estación de Biología Tropical Roberto Franco (EBTRF), Carrera 33 #33–76, Villavicencio, Meta, Colombia Asociación de Becarios de Casanare Yopal Colombia; 4 Asociación de Becarios de Casanare-ABC, Carrera 39#15–35, Yopal, Casanare, Colombia Estación de Biología Tropical Roberto Franco Villavicencio Colombia

**Keywords:** Casanare, Cis-Andean, Cordillera Oriental, diversity, phylogeny, South America, taxonomy

## Abstract

A new species of *Pristimantis* (Craugastoridae, subgenus Pristimantis) is described from a relict and unexplored cloud forest in the western slope from Cordillera Oriental of the Colombian Andes. The specific name was chosen by consensus expert scientists and local people. *Pristimantis chamezensis***sp. nov.** is easily distinguished from congeneric species by having a gray iris with black reticulations in life, subconical tubercles on the upper eyelid, the chin edged with irregular, dark-brown blotches, and conical heel tubercles. The phylogenetic analyses suggest that the origin and radiation of its clade may have occurred in the highlands. With the description of *P. chamezensis***sp. nov.**, we identify 14 species distributed throughout the eastern slope of the Andes that are associated with the Orinoco Basin.

## Introduction

The amphibian fauna from Colombia is among the richest and most diverse in the world (Lynch 1999; Grant et al. 2008) and includes 843 species (Acosta-Galvis 2020). A significant number of these species is grouped in the so-called Terrarana; an unranked taxonomic grouping of at least four closely related families characterized by direct development, egg embryos, and terrestrial reproduction (Hedges et al. 2008; Heinicke et al. 2009, 2018). Terrarana richness in Colombia includes 268 species in 13 genera (Acosta-Galvis 2020), among which the frogs of the *Pristimantis* genus represent the greatest diversity with 83% of the described species.

Morphologically, frogs of the genus *Pristimantis* are easily recognizable among other features by terminal discs on expanded digits and T-shaped terminal phalanges, a dentigerous process of the vomers usually present, and toe IV much longer than toe III (Hedges et al. 2008; Duellman and Lehr 2009). However, this genus still has latent phylogenetic challenges (Navarrete et al. 2016), and recent proposals, based on molecular phylogenies (Hedges et al. 2008; Padial et al. 2014; Páez and Ron 2019; Reyes-Puig et al. 2020), reassigned or excluded members of the species groups from evolutionary arrangements, which were previously based solely on morphological evidence (Lynch and Duellman 1980, 1997).

The genus *Pristimantis* in Colombia is represented by 223 formally described species (Acosta-Galvis 2020). The geographic and ecological complexity of the Andes harbors the greatest richness and rate of endemism in contrast to the lowlands of the Amazon and Pacific basins (Lynch et al. 1997). Current geological evidence of the north-Andean region indicates that the northern formations in Colombia (Occidental, Central, and Oriental mountains ranges) have promoted speciation processes in the genus *Pristimantis* and, therefore, have high diversity and endemism (Lynch and Duellman 1997; Lynch 1999; García-R et al. 2012; Mendoza et al. 2015; Meza-Joya and Torres 2016; Acevedo et al. 2020). Among these geographical units, the Cordillera Oriental contains 44 species, with 13 species inhabiting the Andean and sub-Andean forests on the eastern slopes (Table 1), as part of the Orinoco basin (Acosta-Galvis et al. 2010; Rivera-Correa et al. 2016; Ospina-Sarria and Angarita-Sierra 2020; Acevedo et al. 2020).

**Table 1.** Species of the genus *Pristimantis* from the eastern slope of Cordillera Oriental (Orinoco Basin) in Colombia.

Genus (Subgenus) Species	Species group	Ecoregional distribution	Altitude(m a.s.l)	Reference
Pristimantis (Pristimantis) vilarsi	*conspicillatus* group	sub-Andean, Amazonian and Orinoco.	200–600	Lynch 1975; 1980, 1994; Padial et al. 2014; Heyer and Barrio-Amorós 2009.
Pristimantis (Pristimantis) medemi	*conspicillatus* group	Andean and sub-Andean.	450–2400	Lynch 1994, 2006; Malambo and Marin 2006; Acosta-Galvis et al. 2010; Acosta-Galvis and Alfaro-Bejarano 2011.
Pristimantis (Pristimantis) carranguerorum	*conspicillatus* group	Andean.	1350–2060	Lynch 1994; Renjifo-Rey 2005; Acosta-Galvis and Alfaro-Bejarano 2011; Anganoy-Criollo and Ramírez 2017.
Pristimantis (Hypodictyon) w-nigrum	*ridens* group	Andean and sub-Andean.	800–3000	Cochran and Goin 1970; Lynch and Duellman 1980; Bernal and Lynch 2008.
Pristimantis (Pristimantis) savagei	Unassigned	Andean and sub-Andean.	600–3000	Pyburn and Lynch 1981; Lynch 1994; Ruiz-Carranza et al. 1996; Acosta-Galvis 2000; Lynch 2006; Bernal and Lynch 2008; Acosta-Galvis et al. 2010; Acosta-Galvis and Alfaro-Bejarano 2011.
Pristimantis (Pristimantis) frater	Unassigned	Andean and sub-Andean.	600–3000	Pyburn and Lynch 1981; Lynch 1994; Ruiz-Carranza et al. 1996; Acosta-Galvis 2000; Lynch 2006; Bernal and Lynch 2008; Acosta-Galvis et al. 2010; Acosta-Galvis and Alfaro-Bejarano 2011.
Pristimantis (Pristimantis) bogotensis	Unassigned	Andean, sub-paramos and paramos.	2410–3520	Cochran and Goin 1970; Ruiz-Carranza et al. 1996; Acosta-Galvis 2000; Bernal and Lynch 2008.
Pristimantis (Pristimantis) anolirex	Unassigned	Andean, sub-paramos and paramos.	1800–3550	Lynch 1983; Ardila-Robayo and Acosta-Galvis 2000; Bernal and Lynch 2008.
Pristimantis (Pristimantis) lynchi	Unassigned	Andean, sub-paramos and paramos.	1600–3590	Duellman and Simmons 1977; Bernal and Lynch 2008; Acosta-Galvis 2015.
Pristimantis (Pristimantis) dorado	Unassigned	Andean.	2650	Rivera-Correa et al. 2016
Pristimantis (Pristimantis) terrapacis	Unassigned	sub-Andean	713	Ospina-Sarria and Angarita-Sierra 2020
Pristimantis (Pristimantis) ardilae	*conspicillatus* group	sub-Andean	400–700	Acevedo et al. 2020
Pristimantis (Pristimantis) bowara	Unassigned	sub-Andean	500–665	Acevedo et al. 2020

During field studies along an unexplored cloud forest (2140 m a.s.l.) in the Cordillera Oriental, we collected several specimens of *Pristimantis* that, due to their morphological characters, are not assignable to any described species in this region. Based on the analysis of its molecular data and morphology, we describe a new species recognized by its molecular and morphological distinctiveness.

## Methods

### Study area

**Figure 1.**
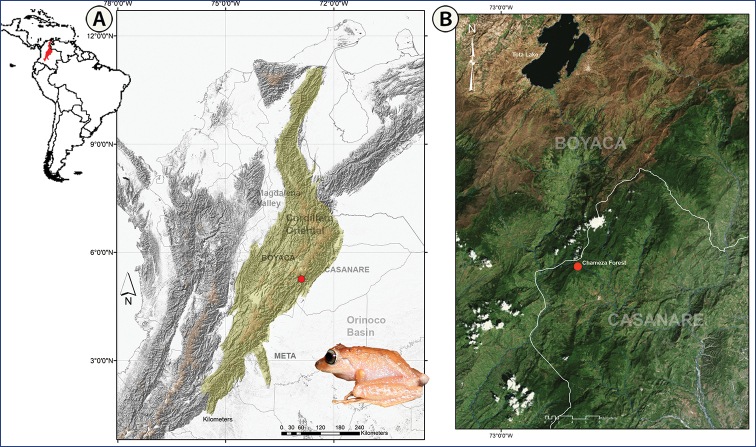
Geographic location in Colombia showing the type locality of *Pristimantis chamezensis* sp. nov. in the western slope of the Cordillera Oriental **A** red dot shows the type locality **B** the landscape of natural pristine forest on the eastern slopes of the Central Cordillera Oriental. Map produced using Arc Map, World Imagery.

**Figure 2.**
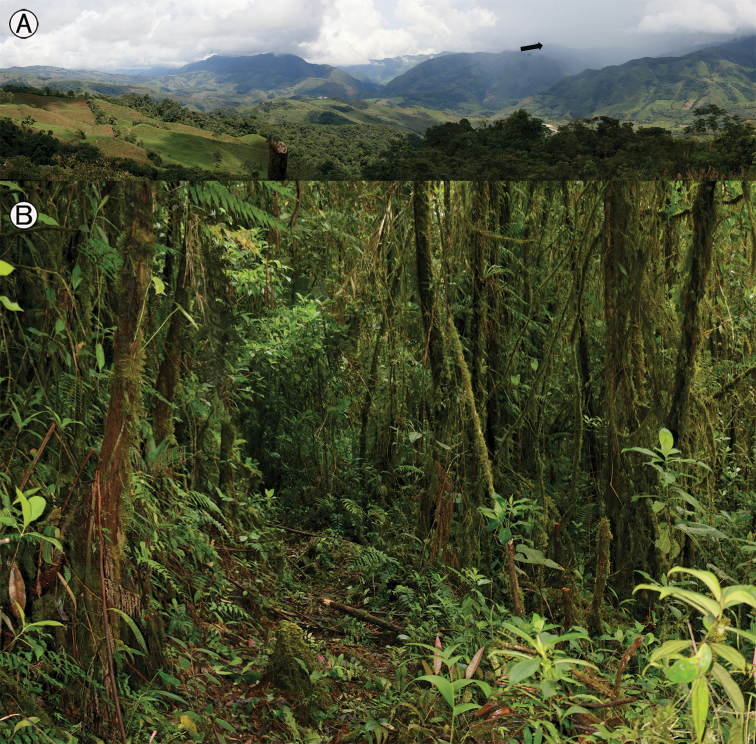
**A** general landscape showing the mountains of the Vereda Centro Norte, Chámeza forest at Cerro Pan de Azúcar (black arrow); type locality of *Pristimantis chamezensis* sp. nov. **B** inside the cloud forests; microhabitat where individuals were found. Photographs by Andrés Acosta-Galvis.

We collected by actively searching from September 2 to November 29, 2010, using intensive visual encounter surveys (Crump and Scott 1994) during evenings in the cloud forests in the municipality of Chámeza (05°15'24.4"N, 072°53'51.6"W), Department of Casanare, Colombia (Fig. 1). This locality is part of an elevated area between 1700–2200 m a.s.l. in an unexplored northern portion of the Cordillera Oriental. This mountainous area consists mainly of pristine natural forests of the Andes orobiome (Fig. 2) within the ecoregion of the Eastern Cordillera montane forests of Colombia (Dinerstein et al. 1995; Olson and Dinerstein 2002). We recorded geographical coordinates and elevations at collecting sites using a Garmin GPSMAP 60CSx (map datum WGS 84).

### Data collection and laboratory procedures

Molecular distinctiveness and phylogenetic relationships of the new species were assessed by analyzing DNA sequences of mitochondrial DNA (mtDNA) which included a fragment of the 16S ribosomal RNA (16S) and a fragment of the cytochrome oxidase subunit 1 (COI) genes. We assembled a data set that included only the 16S gene fragment by aligning sequences from all known *Pristimantis* species from the eastern slopes of the Cordillera Oriental of Colombia together with the most similar sequences already published in Genbank (Table 2). For this, we conducted a search for sequences similar to the 16S gene fragment of the new species using the BLAST algorithm in GenBank. The most similar 127 BLAST hits to the sequences from the new species were downloaded, aligned, and assessed using Bayesian (BA) and maximum likelihood (ML) analyses. After removing distant and redundant sequences, the final dataset contained 58 sequences of 827 base pairs (bp) of the 16S, including the new species and *Pristimantis medemi* (Lynch, 1994) obtained in this study (Table 1). We assembled a complete data set comprising sequences of the 16S, concatenated with sequences of the COI gene for a subset, including the new species and its following six most-related species, selected based on the results of the analyses: *Pristimantis carranguerorum* (Lynch, 1994), *P. bowara*Acevedo et al., 2020, *P. lutitus* (Lynch, 1984), *P. medemi* (Lynch, 1994), *P. nicefori* (Cochran & Goin, 1970), and *P. savagei* (Pyburn & Lynch, 1981).

**Table 2.** Species of *Eleutherodactylus*, *Pristimantis*, and GenBank accession numbers of the DNA sequences used in the phylogenetic analyses.

Species	Accession numbers		Voucher code	Source
	16S rRNA	COI		
*E. johnstonei*	EF493561	–	USNM336018	Heinicke et al. 2007
*P. acatallelus*	JN371032	–	UVC:15863	García-R et al. 2012
*P. achatinus*	EF493660	–	KU217809	Heinicke et al. 2007
*P. achatinus*	JN104676	–	UVC:15867	García-R et al. 2012
*P. aniptopalmatus*	EF493390	–	KU291627	Heinicke et al. 2007
*P. bipunctatus*	EF493702	–	KU291638	Heinicke et al. 2007
*P. bogotensis*	JN991432	JN991362	NRPS003	Pinto-Sanchez et al. 2012
*P. bowara*	MN215434	–	MCNUPH304	Acevedo, Armesto and Palma 2000
*P. buccinator*	KY652650	–	MUSM:33269	von May et al. 2017
*P. buckleyi*	EF493350	–	KU217836	Heinicke et al. 2007
*P. caprifer*	EF493391	–	KU177680	Heinicke et al. 2007
*P. carranguerorum*	KP149324	KP149128	LSB385	Guarnizo et al. 2015
*P. chamezensis* sp. nov.	MK776946	MK789293	ARA5848	This study
*P. chamezensis* sp. nov.	MK776947	MK789294	ARA5849	This study
*P. citriogaster*	EF493700	–	KU212278	Heinicke et al. 2007
*P. condor*	EF493701	–	KU217857	Heinicke et al. 2007
*P. conspicillatus*	EF493529	–	QCAZ28448	Heinicke et al. 2007
*p. curtipes*	EF493513	–	KU217871	Heinicke et al. 2007
*P. devillei*	EF493688	–	KU217991	Heinicke et al. 2007
*P. dorado*	KU496877	–	MRC636	Rivera-Correa et al. 2016
*P. duellmani*	AY326003	–	WED 53050	Darst and Cannatella 2004
*P. fenestratus*	EF493703	–	–	Heinicke et al. 2007
*P. frater*	KP149461	–	AJC 4015	Guarnizo et al. 2015
*P. gentryi*	EF493511	–	KU218109	Heinicke et al. 2007
*P. koehleri*	EU192279	–	MNKA 6627	Padial and De la Riva 2009
*P. lasalleorum*	KY494221	–	ICN55758	González-Durán et al. 2017
*P. latro*	MK174413	–	LZA 1340	Cornelio et al. unpublished
*P. leptolophus*	KY494226	–	JJS093	González-Durán et al. 2017
*P. lutitus*	KP149401	KP149196	AJC3490	Guarnizo et al. 2015
*P. lymani*	EF493392	–	KU218019	Heinicke et al. 2007
*P. maculosus*	KY494240	–	ICN55760	González-Durán et al. 2017
*P. malkini*	EU186663	–	QCAZ28296	Hedges et al. 2008
*P. medemi*	MK776948	MK789295	ARA2655	This study
*P. nicefori*	MN215436	MN218387	MCNUPH48	Acevedo, Armesto and Palma 2000
*P. parectatus*	KY627810	–	MHUAA9977	Rivera-Correa et al. 2017
*P. peraticus*	KY494224	–	WB1301	González-Durán et al. 2017
*P. peruvianus*	EF493707	–	–	Heinicke et al. 2007
*P. quinquagesimus*	EF493690	–	KU179374	Heinicke et al. 2007
*P. rhabdolaemus*	EF493706	–	KU173492	Heinicke et al. 2007
*P. sagittulus*	EF493705	–	KU291635	Heinicke et al. 2007
*P. samaipatae*	EU192290	–	MNCN 42988	Padial and De la Riva 2009
*P. savagei*	KP149382	KP149180	AJC3995	Guarnizo et al. 2015
*P. scoloblepharus*	KY494236	–	ICN55768	González-Durán et al. 2017
*P. skydmainos*	EF493393	–	–	Heinicke et al. 2007
*P. simonbolivari*	EF493671	–	KU218254	Heinicke et al. 2007
*P. simonsii*	EU186665	–	KU212350	Hedges et al. 2008
*P. surdus*	EF493687	–	KU177847	Heinicke et al. 2007
*P.* sp.1	KY494239	–	JJS122	González-Durán et al. 2017
*P.* sp.2	KY494238	–	ICN55759	González-Durán et al. 2017
*P.* sp.3	KY494230	–	ICN55756	González-Durán et al. 2017
*P.* sp.4	KY494234	–	ICN55774	González-Durán et al. 2017
*P.* sp.5	KY494223	–	ICN55775	González-Durán et al. 2017
*P. stictogaster*	EF493704	–	KU291659	Heinicke et al. 2007
*P. thymalopsoides*	EF493514	–	KU177861	Heinicke et al. 2007
*P. toftae*	EF493353	–	KU215493	Heinicke et al. 2007
*P. unistrigatus*	EF493387	–	KU218057	Heinicke et al. 2007
*P. uranobates*	KY494223	–	ICN55787	González-Durán et al. 2017
*P. vertebralis*	EF493689	–	KU177972	Heinicke et al. 2007
*P. vilarsi*	KP149391	KP149187	AJC2113	Guarnizo et al. 2015

From two tissue samples of the new species and a tissue sample of *Pristimantis medemi* we extracted total genomic DNA using a standard Phenol-Chloroform method (Sambrook et al. 1989). We amplified the gene fragments using the primers pairs 16Sbr-H/16SC-16L (Palumbi et al. 1991; Darst and Cannatella 2004, respectively) and LCO1490/HCO2198 (Folmer et al. 1994) for the 16S and COI, respectively. We carried out PCRs in a total volume of 30 μl containing one unit Taq polymerase (Bioline; Randolph, MA), 1× of a buffer (Bioline), a final concentration of 1.5 mM MgCl2 (Bioline), 0.5 μM of each primer, 0.2 mM of each dNTP (Bioline), 0.2 µg of bovine serum albumin (BSA), and approximately 50 ng of total DNA. We purified the PCR products using the ammonium acetate protocol (Bensch et al. 2000), and we sequenced them on an ABI 3130xl Genetic Analyzer (Applied Biosystems, Foster City, CA, USA) using the BigDye Terminator v. 3.1 Cycle Sequencing Kit (Applied Biosystems). We stored the remaining DNA extractions at –80 °C in the tissue collection of the Instituto de Genética, Universidad Nacional de Colombia (for voucher numbers see Table 2). We performed the thermocycling conditions as indicated by the authors, who reported the primers for the obtained fragments. The GenBank accession numbers of the obtained sequences are MK776946–MK776948 and MK789293–MK789295. We edited and aligned the sequences using Chromas 1.51 (http://www.technelysium.com.au/chromas.html) and BioEdit v. 7.0.5.2 (Hall 1999). To exclude divergent regions and poorly aligned bases from the 16S dataset, we used the software Gblocks v. 0.91b (Castresana 2000; Talavera and Castresana 2007; available as a web server at http://molevol.cmima.csic.es/castresana/Gblocks server.html), which resulted in a final alignment of 528 base pairs (bp). The COI alignment consisted of 652 bp.

### Phylogenetic and genetic divergence analyses

We analyzed the complete evidence dataset using the following partition scheme: (i) unpartitioned; (ii) partitioned by gene (i.e., each gene fragment treated as a distinct partition); and (iii) maximum partitioning (i.e., we treated each codon of the protein-coding gene COI and the ribosomal gene fragment as distinct partitions). We assessed the optimal partitioning scheme and best-fit evolutionary models using PartitionFinder v. 1.1.1 and the Bayesian Information Criterion (Lanfear et al. 2012), resulting in the selection of the maximum partitioning scheme. For the 16S dataset, the obtained model (SYM + G) was applied in a Bayesian analysis (BA) with MrBayes v. 3.2.1 (Ronquist et al. 2012). For the complete evidence dataset, we applied the 16S fragment model plus the following complementary COI fragment resulting models in a Bayesian analysis with MrBayes: COI 1^st^ codon – TrNef + G, COI 2^nd^ codon – HKY, COI 3^rd^ codon – HKY. We incorporated these models into a single tree search (mixed model partition approach; Nylander et al. 2004). For both analyses, we carried out two parallel runs using four Markov chains, each starting from a random tree. We ran the Markov chains for 10 million generations. The burn-in was set to sample only the plateau of the most likely trees that were used for generating a 50% majority rule consensus. We used the software TRACER v. 1.5.4 (Rambaut and Drummond 2007) to assess an acceptable level of the MCMC chain mixing and to estimate effective sample sizes for all parameters. Additionally, maximum likelihood (ML) analyses were run using RAxML 7.2.8 (Stamatakis 2006) and the GTR+G model. We performed five independent maximum likelihood searches with different starting conditions and the rapid bootstrap algorithm to explore the robustness of the branching patterns by comparing the best trees. Afterward, 1000 non-parametric thorough bootstrap values were computed and plotted against the best tree. The Genbank sequence of *Eleutherodactylus johnstonei* Barbour, 1914, EF493561, was used as outgroup. To assess the genetic divergence between the new and the other *Pristimantis* species, we calculated uncorrected *p* genetic distances for the 16S and the COI fragments using MEGA v. 7.0.21 (Kumar et al. 2016).

### Morphology

We euthanized specimens using Clorethone, which were then fixed in 10% formalin, preserved in 70% ethanol, and deposited in the biological collections of the Instituto de Investigación de Recursos Biológicos Alexander von Humboldt, Villa de Leyva, Boyacá, Colombia (IAvH-Am). Other specimens examined are listed in Suppl. material 1. The criteria for the definition of descriptions and diagnostic characters followed Duellman and Lehr (2009), Lynch and Duellman (1997), and Navarrete et al. (2016). To identify sex and sexual maturity, we made a small incision in the groin region for macroscopic observation of the gonads. Adult males have the granular testis, while females show enlarged, thickened, and convoluted oviducts. Morphometric measurements were made with digital calipers (nearest 0.01 mm) or a Nikon stereoscopic microscope SMZ-1B with high Intensity Illuminator NI-150 Nikon as follows: SVL (snout-vent length), HW (head width), HL (head length from the tip of the snout to the posterior border of the skull, posterior edge of prootic, noted through the skin), IOD (interorbital distance), ED (eye diameter), EN (eyes-nares distance), UEW (upper eyelid width), ETS (distance between the anterior edges of the eye to the tip of the snout), TD (horizontal tympanum diameter), RW (rostral width), InD (internarial distance), TL (tibial Length), FL (femur length), FtL (foot length), and HnL (hand length). Means are reported ± one standard error. We photographed habitats and specimens using Canon EOS 30D and EOS 5D Mark II digital cameras inside a Photo Safe-box using 5.500 kelvins LED lights.

## Results

### Phylogenetic and genetic divergence analyses

**Figure 3.**
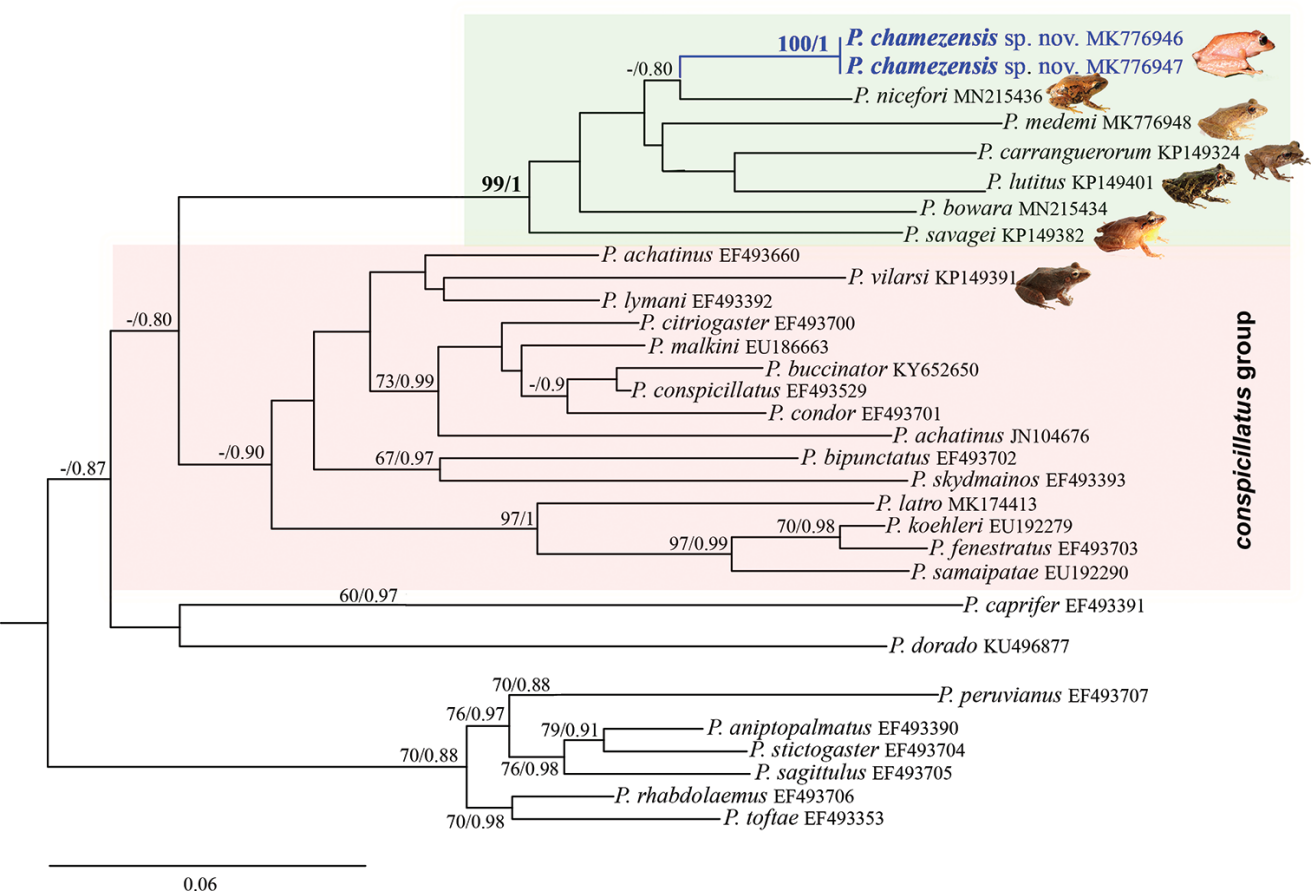
Maximum likelihood inference tree showing the evolutionary relationships of *Pristimantis chamezensis* sp. nov. (bold) and its 28 more closely related *Pristimantis* species based on 528 bp of the 16S rRNA gene. Numbers before nodes: thorough maximum likelihood (ML) bootstrap percentages left and Bayesian analysis (BA) posterior probability values right. Bootstrap values below 50% and posterior probabilities below 0.5 not shown. Outgroup taxon removed.

The resulting phylogenetic tree including all 58 sequence of the 16S fragment is shown in the Suppl. material 2: Fig. S1. A reduced phylogenetic tree including the 16S fragment sequences of the new species and its closest 29 sequences is shown in Figure 3. The following description is referring to the reduced tree. Based on the phylogenetic relationships, the new species could be assigned to the genus *Pristimantis*, subgenus Pristimantis. Both tree-building methods revealed *Pristimantis chamezensis* sp. nov. with maximum support within a supported monophyletic group comprising *Pristimantis carranguerorum*, *P. bowara*, *P. lutitus*, *P. medemi*, *P. nicefori*, and *P. savagei* (Fig. 3). Both analyses concurred in placing the new species as a sister taxon of *P. nicefori* with low support (ML: 40%; BA: 0.80). The other 23 *Pristimantis* species were revealed by both analyses within three separated, weakly supported clades, exhibiting low supported evolutionary relationships (Fig. 3). For the complete evidence dataset, both tree building methods revealed *Pristimantis chamezensis* sp. nov., as part of a monophyletic clade also comprising *P. carranguerorum*, *P. bowara*, *P. lutitus*, *P. medemi*, *P. nicefori*, and *P. savagei* with maximum support (Suppl. material 3: Fig. S2). Both analyses revealed that the new species is the sister taxon of a clade showing the following weakly supported phylogenetic relationships: (((*P. lutitus* + *P. bowara*) *P. nicefori*) *P. carranguerorum*). Finally, *P. medemi* and *P. savagei* were revealed as successive sister taxa of the that clade plus the new species, with low support (Suppl. material 3: Fig. S2). Genetic distances for the 16S gene between *P. chamezensis* sp. nov. and *P. nicefori*, *P. carranguerorum*, and *P. savagei* were 4.8%, 5.2%, and 5.9%, respectively. Distances between *P. chamezensis* sp. nov. and *P. medemi*, *P. lutitus*, and *P. bowara* were 6.2%, 6.2%, and 6.7%, respectively (Table 3). The sequence divergence range of the monophyletic group compared to the other analyzed taxa was 5.9–4.1% (Table 3). The uncorrected *p* distances for the COI gene revealed that sequence differentiation values between *P. chamezensis* sp. nov. and *P. carranguerorum*, *P. nicefori*, *P. lutitus*, *P. savagei*, and *P. medemi* were 6.2%, 6.4%, 6.7%, 6.7%, and 6.7%, in that order. For the same gene fragment, the distance between *P. chamezensis* sp. nov. and *P. bowara* was 7.8%.

**Table 3.** Uncorrected *p*-distances for the fragment of 16S gene (528 bp) of the *Pristimantis* species, expressed as percentages (means).

	n	*cha*	*nic*	*car*	*sav*	*med*	*lut*	*bow*	*cit*	*mal*	*con*	*buc*	*lym*	*ach*	*con*	*sti*	*rha*	*cap*	*ach*	*tof*	*plu*	*ani*	*bip*	*lat*	*koe*	*fen*	*sam*	*sag*	*vil*	*dor*	*sky*
*chamezensis* sp. nov.	**2**	0.0																													
* nicefori * MN215436	1	4.8	–																												
* carranguerorum * KP149324	1	5.2	4.8	–																											
* savagei * KP149382	1	5.9	5.2	5.5	–																										
* medemi * MK776948	1	6.2	5.9	6.2	5.0	–																									
* lutitus * KP149401	1	6.2	6.2	6.9	5.9	7.6	–																								
* bowara * MN215434	1	6.7	6.2	6.4	4.0	5.7	6.9	–																							
* citriogaster * EF493700	1	7.8	6.7	6.2	5.9	6.4	7.1	6.4	–																						
* malkini * EU186663	1	8.3	7.8	8.6	9.0	8.8	10.2	9.2	10.0	–																					
* conspicillatus * EF493529	1	8.3	8.3	9.3	9.5	8.8	10.0	10.0	10.2	3.1	–																				
* buccinator * KY652650	1	8.3	8.3	9.7	8.8	9.3	9.7	10.4	10.0	2.8	2.4	–																			
* lymani * EF493392	1	8.3	8.3	10.5	8.3	8.8	9.7	10.0	10.9	4.0	3.8	2.1	–																		
* achatinus * EF493660	1	8.8	8.3	9.0	7.8	8.8	9.5	9.0	9.7	3.8	4.7	3.8	5.0	–																	
* condor * EF493701	1	8.8	8.8	9.8	9.3	10.0	10.2	10.5	11.7	5.2	5.0	4.8	5.9	4.3	–																
* stictogaster * EF493704	1	9.0	8.8	9.7	9.2	9.7	9.5	10.0	10.7	4.0	3.3	2.8	4.5	4.7	5.5	–															
* rhabdolaemus * EF493706	1	9.0	9.0	9.8	9.7	8.8	10.9	10.5	9.8	9.7	9.5	9.3	10.2	9.5	9.8	9.7	–														
* caprifer * EF493391	1	9.1	9.0	9.5	9.0	8.1	9.7	9.7	9.7	9.7	9.7	9.2	10.2	9.7	9.3	9.2	3.1	–													
* achatinus * JN104676	1	9.2	9.1	9.1	9.3	9.1	10.3	9.8	9.1	9.3	8.6	8.4	9.5	8.4	7.9	9.5	9.3	8.6	–												
* toftae * EF493353	1	9.2	9.2	10.2	10.9	11.9	11.1	11.1	13.1	7.1	7.3	6.9	7.6	7.8	7.6	5.9	10.9	10.7	11.2	–											
* aniptopalmatus * EF493390	1	9.2	9.2	10.5	10.7	10.0	10.7	10.9	9.7	10.9	10.4	10.7	11.6	10.9	11.6	10.7	3.3	4.3	10.3	11.8	4.3	–									
* bipunctatus * EF493702	1	9.5	9.2	9.5	9.5	9.0	11.1	10.2	9.5	9.7	10.0	9.2	10.2	9.2	10.5	10.2	2.4	3.1	9.5	11.1	3.6	2.8	–								
* latro * MK174413	1	10.0	9.5	9.8	9.0	10.2	10.2	9.5	11.0	6.9	6.4	6.2	6.9	6.4	7.1	5.7	10.0	10.7	9.3	7.8	10.5	10.5	10.2	–							
* koehleri * EU192279	1	10.0	10.0	11.5	10.5	9.4	12.2	10.8	11.3	7.9	7.7	8.6	9.8	9.1	8.6	8.4	9.6	9.3	9.4	11.2	9.8	11.0	10.0	9.4	–						
* fenestratus * EF493703	1	10.0	10.0	11.4	11.4	10.2	11.1	11.6	11.6	7.8	8.1	8.8	9.2	7.6	8.6	7.6	10.9	10.4	10.0	8.5	10.4	11.8	11.1	8.6	6.9	–					
* samaipatae * EU192290	1	10.4	10.0	11.9	11.4	10.0	10.7	11.6	11.6	8.3	7.6	8.8	9.2	7.8	8.8	7.6	11.2	10.2	10.0	8.8	10.2	12.1	10.9	9.3	6.7	1.2	–				
* sagittulus * EF493705	1	11.1	10.4	10.5	10.2	9.7	10.2	10.9	10.2	8.1	7.6	7.6	8.8	7.6	8.6	7.8	10.5	10.2	10.0	10.2	10.9	11.1	9.7	9.3	7.2	3.3	3.6	–			
* vilarsi * KP149391	1	11.4	11.1	11.4	10.2	10.2	12.1	11.1	10.5	11.6	11.4	11.1	11.6	11.1	11.4	11.6	3.6	4.0	10.7	13.0	4.3	4.5	3.1	11.6	11.0	12.1	11.8	11.1	–		
* dorado * KU496877	1	11.4	11.4	10.0	9.5	10.0	12.1	11.2	11.4	8.1	7.6	7.8	8.6	7.6	7.6	7.8	11.2	10.9	10.8	10.9	12.8	13.1	11.4	9.0	9.1	10.2	10.5	9.3	12.6	–	
* skydmainos * EF493393	1	11.6	11.4	13.1	11.4	14.1	13.6	12.8	14.1	9.6	9.6	8.9	9.4	11.4	11.9	9.6	12.1	13.3	12.7	12.6	13.8	13.8	12.6	10.4	11.2	11.9	11.9	11.6	14.1	12.8	–

### Description of new species

#### Pristimantis chamezensis sp. nov.

Taxon classificationAnimaliaAnuraStrabomantidae

0231086A-DB3D-509A-9F8A-15F37A8C8F83

http://zoobank.org/ff99cfe4-4fa7-402b-8d76-d921a93d1566

**Figure 4.**
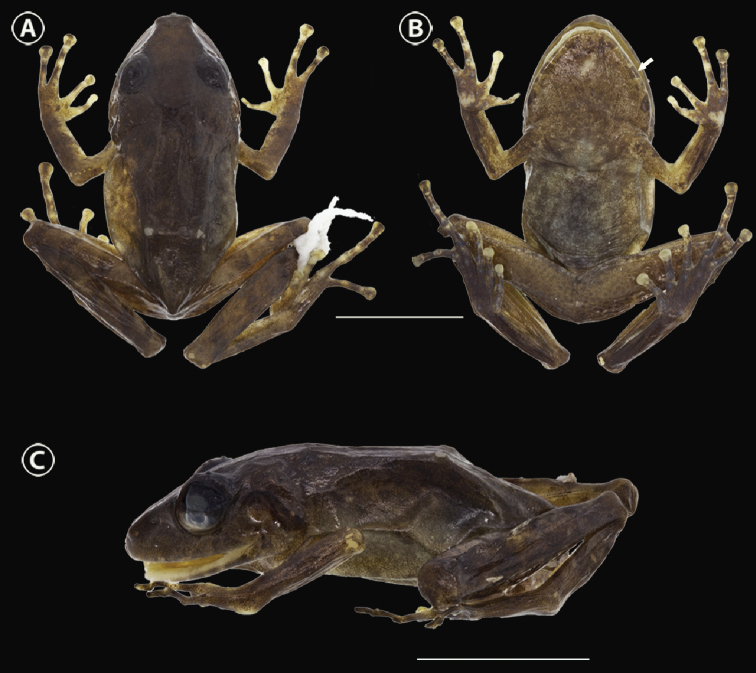
*Pristimantis chamezensis* sp. nov. preserved holotype, adult female, IAvH-Am-10269 (SVL = 23.8 mm) **A** dorsal view **B** ventral view **C** lateral view. White arrow = chin with irregular blotches of dark brown. Scale bar: 10 mm. Photographs by Andrés Acosta-Galvis.

**Figure 5.**
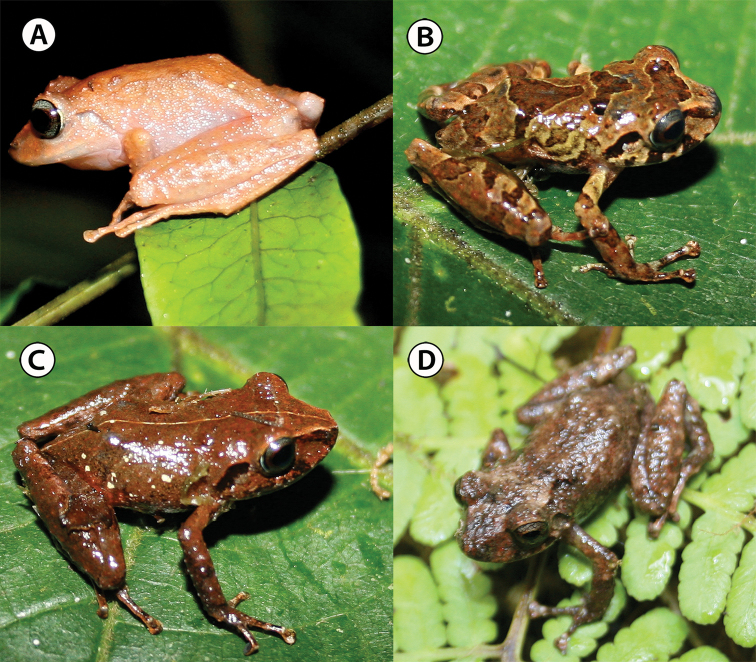
*Pristimantis chamezensis* sp. nov., live specimens. **A** Holotype, adult female, IAvH-Am-10269 (SVL= 23.8 mm) **B** juvenile, IAvH-Am-10283 (SVL = 17.5 mm) **C** paratype, adult female, IAvH-Am-10277 (SVL = 19.7 mm) **D** paratype, adult male, IAvH-Am-10267 (SVL = 22.6 mm). Photographs by Andrés Acosta-Galvis.

Figs 4 , 5 ; Table 4

##### Holotype.

IAvH-Am-10269 (field number ARA 5852. Figs 4, 5) an adult female collected on 3 September 2010 by Andrés R. Acosta-Galvis, Beatriz Ramirez, José Pérez, Luis Daniel Prada, and Natalia Novoa.

##### Type locality

(Figs 1, 2). Colombia, Casanare Department, Chámeza Municipality, vereda Centro Norte, Chámeza forest, Cerro Pan de Azúcar, eastern flank of the Cordillera Oriental, Colombia. 05°15'24.4"N, 072°53'51.6"W, 2140 m a.s.l.

##### Paratypes

**(11)** (Fig. 5; Table 4). IAvH-Am-10267, IAvH-Am-10270–10274, adult males; IAvH-Am-10275–10277, IAvH-Am-10282, adult females, collected on 13 November 2010 by Andrés R. Acosta-Galvis, Beatriz Ramirez, José Pérez, Luis Daniel Prada, and Natalia Novoa; same locality as the holotype.

##### Referred specimens.

IAvH-Am-10268, IAvH-Am-10278–10281, IAvH-Am-10283–10287, juveniles, same locality and date as paratypes.

##### Diagnosis

**Figure 6.**
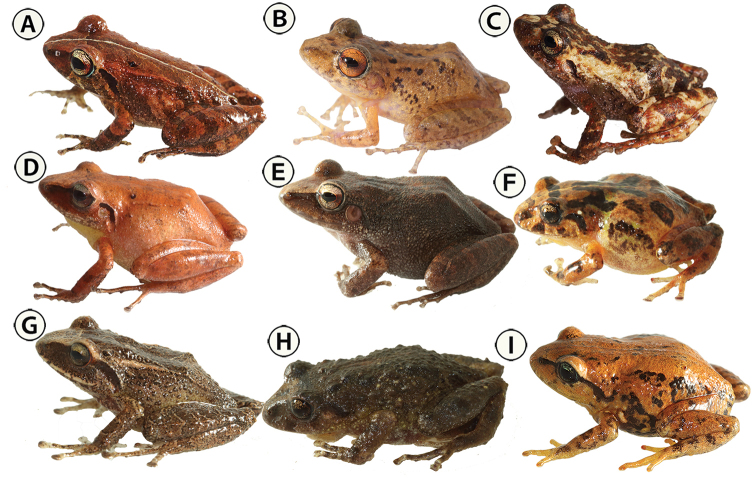
Live specimens (lateral view) of *Pristimantis* currently known from the eastern Andean Cordillera associated with the Orinoco basin in Colombia. **A***Pristimantis carranguerorum*, Medina Municipality, Cundinamarca Department, IAvH-Am-14954 (adult female, SVL = 22.6 mm) **B***Pristimantis medemi*, Medina Municipality, Cundinamarca Department, IAvH-Am-15025 (adult male, SVL = 32.5 mm) **C***Pristimantis frater*, Medina Municipality, Cundinamarca Department, IAvH-Am-14923 (adult female, SVL = 27.8 mm) **D***Pristimantis savagei*, Medina Municipality, Cundinamarca Department, IAvH-Am-14933 (adult male, SVL = 23.2 mm) **E***Pristimantis vilarsi*, La Macarena Municipality, Meta Department, IAvH-Am-15095(Adult female, SVL = 44.8 mm) **F***Pristimantis bogotensis*, Cabrera Municipality, Cundinamarca Department, IAvH-Am-15345 (adult male, SVL = 21.9 mm) **G***Pristimantis anolirex*, Santa Barbara Municipality, Santander Department, IAvH-Am-15654 (juvenile female, SVL = 22.3 mm) **H***Pristimantis lynchi*, Tasco Municipality, Boyacá Department, IAvH-Am-15871 (adult male, SVL = 22.1 mm) **I***Pristimantis nicefori*, Santa Barbara Municipality, Santander Department, IAvH-Am-15730 (SVL = 24.5 mm). Photographs by Andrés Acosta-Galvis.

**Figure 7.**
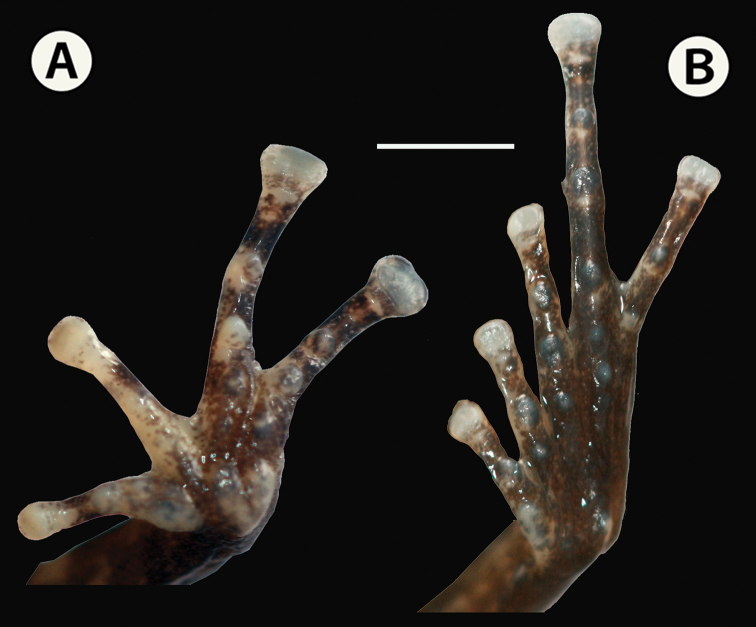
Hand and toes of adult male paratype, IAvH-Am-10271 of *Pristimantis chamezensis* sp. nov. in ethanol 70%. **A** Ventral view of foot **B** ventral view of hand. Scale bar: 2 mm. Photographs by Andrés Acosta-Galvis.

(Figs 4–7). A species of *Pristimantis* characterized by the following combination of morphological characters: (1) dorsal skin shagreen with scattered larger tubercles; dorsolateral folds absent; discoidal fold visible; skin on venter areolate. (2) Tympanic membrane and tympanic annulus present, its dorsoposterior border converges with supratympanic fold; its diameters are 35.6–56.0% of the eye diameter; small, barely visible, subconical postrictal tubercles. (3) Snout short, broadly rounded in dorsal view and rounded in lateral view; *canthus rostralis* sharp and concave. (4) Upper eyelid bearing one to three subconical tubercles, narrower than IOD. (5) Choanae small, subovoid; dentigerous processes of vomers prominent, oblique, and widely separated from each other, bearing 8 or 9 teeth. (6) Males with vocal slits; subgular vocal sac observable; nuptial pads not evident. (7) Finger I shorter than II, with discs expanded and rounded; bifid palmar tubercle. (8) Fingers bearing narrow lateral fringes. (9) Ulnar tubercles absent. (10) Tarsal tubercles present, subconical; heel tubercles present but nearly inconspicuous and conical. (11) Two metatarsal tubercles, with inner tubercle elongate, three times the length of the rounded and prominent outer tubercle; supernumerary plantar tubercles numerous, enlarged, and rounded. (12) Toes with lateral fringes and broad discs; toe V much longer than toe III (disc on toe III extends to the proximal edge of the medial subarticular tubercle on toe IV, disc on toe V extends beyond the distal edge of the penultimate subarticular tubercle on toe IV); webbing absent. (13) Dorsal surface pattern variable, with homogeneous color brown (with or without paravertebral line) or inverted V-shaped markings with dark brown blotches edged with pale cream; iris gray, medially reddish, with black reticulations; ventral surfaces cream-colored to light brown, finely peppered with irregular, diffuse, dark-brown reticulations or blotches; posterior surface of thighs brown; dark-brown labial bars present or absent; edge of the chin with irregular blotches of dark brown (Fig. 5). (14) apparently sexually dimorphic in size (Table 4), with an SVL in adult males 19.6–23.7 mm and 19.0–24.9 mm in adult females.

**Table 4.** Morphometric (in mm) of the type series of *Pristimantis chamezensis* sp. nov. Abbreviations are given in Methods.

IAvH–Am	Sex	SVL	HW	HL	IOD	ED	EN	UEW	ETS	TD	FL	FtL	InD	RW	TL	HnL
10267	M	22.6	7.9	10.1	3.1	3.1	3.0	1.8	4.1	1.7	10.6	10.9	2.5	3.2	12.5	6.4
10271	M	23.7	9.2	9.1	3.6	2.8	3.0	2.0	4.6	1.6	12.6	10.8	2.5	3.8	12.6	6.5
10273	M	20.9	7.5	7.6	2.8	2.8	2.4	2.1	3.7	1.1	10.9	9.6	2.5	3.6	11.6	5.9
10270	M	19.6	7.9	8.9	2.6	2.6	2.8	1.7	3.5	1.1	10.6	9.9	2.3	2.7	11.7	6.1
10274	M	21.2	8.3	9.4	2.8	2.9	3.1	1.8	4.0	1.2	10.5	9.9	2.4	3.3	11.8	5.9
10272	M	20.3	8.0	9.3	3.0	2.7	2.4	1.8	3.7	1.0	10.0	10.1	2.5	2.8	12.2	6.3
Means		21.4	8.1	9.1	3.0	2.8	2.8	1.9	3.9	1.3	10.9	10.2	2.5	3.2	12.1	6.2
Standard error		1.4	0.5	0.8	0.3	0.2	0.3	0.1	0.4	0.3	0.8	0.5	0.1	0.4	0.4	0.2
10276	F	24.9	11.0	11.3	3.4	3.5	3.1	2.6	4.7	1.2	14.3	12.9	3.1	3.8	14.4	8.4
10277	F	19.7	8.1	9.0	2.9	2.8	2.4	1.6	4.1	1.0	11.0	8.8	2.5	2.7	12.1	6.0
10269	F	23.8	9.8	10.4	3.8	3.3	3.2	2.1	4.5	1.1	13.6	12.1	2.9	3.9	14.5	7.4
10275	F	19.0	7.8	9.0	2.9	2.4	2.8	1.7	4.1	1.0	9.7	9.4	2.1	3.1	11.5	5.6
Means		21.9	7.9	8.5	2.9	2.6	2.6	1.8	3.8	1.1	10.6	9.6	2.3	3.1	11.5	6.8
Standard error		2.9	1.4	1.1	0.4	0.4	0.3	0.4	0.2	0.1	2.1	1.9	0.4	0.5	1.5	1.3

##### Species comparisons

(Figs 5–7, Suppl. material 1). The new species is compared to other species of *Pristimantis* in the eastern slope of the Cordillera Oriental in the Orinoco basin of Colombia. The character states of the compared species are enclosed in parentheses. *Pristimantis chamezensis* is distinguished from *P. carranguerorum* by the absence of short dorsolateral folds in the scapular region (present); snout rounded in dorsal view (subacuminate; Fig. 6); the dorsum brown, with some lighter and diffuse reticulations (pale dorsolateral lines; Fig. 6); and subconical tubercles on the upper eyelid (absent). The new species differs from *P. vilarsi* (Melin, 1941) in having the posterior surfaces of the thighs brown in life (reddish); adult females smaller, SVL 19.0–24.9 mm (SVL 25.4–43.2 mm); and the snout broadly rounded in dorsal view (subacuminate). *Pristimantis chamezensis* can be easily confused with *P. savagei* by the presence of one to three subconical tubercles on the upper eyelid; however, it differs by the absence of ulnar tubercles (present); snout broadly rounded in dorsal view (subacuminate); and posterior surface of thighs brown in life (pale orange). *Pristimantis chamezensis* is distinguished from *P. medemi* by having subconical tubercles on the upper eyelids (absent); dorsal and ventral iris gray in life (Fig. 5), medially reddish, with black reticules (orange to yellow); and smaller size, SVL 19.6–26.4 mm (SVL 29.4–43.1 mm). *Pristimantis chamezensis* differs from *P. anolirex* (Lynch, 1983) (Fig. 6) in lacking dorsolateral folds (present on half of the body); ulnar tubercles absent (present and small; Fig. 7); and snout broadly rounded in dorsal view (subacuminate). *Pristimantis chamezensis* is distinguished from *P. lynchi* (Duellman & Simmons, 1977) in having the edge of the chin with irregular blotches (Fig. 4) of dark brown (uniformly brown); palmar tubercle bifid (elliptical); and snout broadly rounded in dorsal view (subacuminate). Compared to *P. bogotensis* (Peters, 1863) (Fig. 6), *P. chamezensis* has a prominent dentigerous process on the vomers, oblique and widely separated from each other (concealed in the palatine tissue); and broadly rounded snout in dorsal view (rounded). *Pristimantis chamezensis* differs from *P. frater* (Werner, 1899) (Fig. 6) in having a broadly rounded snout in dorsal view (acuminate); and toes IV and V with narrow discs (broader). *Pristimantis chamezensis* is distinguished from *P. terrapacis* Ospina-Sarria & Angarita-Sierra, 2020 by having subconical tubercles on upper eyelid and heel (absent) and webbing absent between the toes (basal webbing). *Pristimantis chamezensis* differ from *P. ardilae*Acevedo et al. 2020 by the absence of short dorsolateral folds in the scapular region (present); broadly rounded snout in dorsal view (subacuminate); and upper eyelid with subconical tubercles (without tubercles). *Pristimantis chamezensis* is distinguished from *P. bowara* in having the broadly rounded snout in dorsal view (subacuminate) and dorsal skin shagreen with scattered larger tubercles (smooth). Lastly, *P. chamezensis* that can be distinguished from *P. nicefori* (Fig. 6) in having the discs of the digits expanded (slightly expanded), snout broadly rounded in dorsal view (acuminate), and snout broadly rounded in lateral view (pointed).

##### Description of the holotype.

An adult female (Figs 4, 5) with a snout-vent length (SVL) of 23.8 mm; the skin of cephalic region, dorsum, eyelids, lateral surfaces, and dorsal thighs shagreen with scattered larger tubercles; dorsolateral folds absent and discoidal folds visible; skin on venter areolate. Head length (HL), diagonally from the corner of mouth to tip of snout 10.4 mm; head width (HW) 9.8 mm, approximately equal to width of the body and 41.1% of the SVL. Snout broadly rounded in dorsal view (type F, *sensu*Duellman and Lehr 2009; Fig. 4) and rounded in lateral view (type A, *sensu*Duellman and Lehr 2009; Fig. 4); internarial distance (between center of naris) 2.9 mm; nostril moderately protuberant, directed dorsolaterally; *canthus rostralis* well defined; loreal region slightly concave; lips not prominent. Eye diameter (ED) from its posterior to anterior corner 3.3 mm; its length 73.3% of the ETS (distance between the anterior edge of the eye to the tip of snout); interorbital region wider than upper eyelid; the upper eyelid width (UEW) 55.2% of interorbital distance (IOD); upper eyelid bearing three smaller subconical tubercles (Figs 4, 5); no cranial crests. Supratympanic fold low and short. Tympanic membrane and tympanic annulus present, small, and rounded (Figs 4, 5), its dorsoposterior border converges with supratympanic fold; its diameter 1.1 mm and equivalent to 33% of eye diameter (ED). Choanae subovoid, not concealed by the palatal shelf of the maxillary arch; dentigerous processes of vomers prominent, nine teeth positioned posterior to level of choanae and widely separated from each other. Tongue rounded, its posterior border notched for half of its extension is adherent to the floor of mouth; teeth present on the maxillary arch.

Forelimbs of moderate size, forearm length 6.4 mm; ulnar tubercles absent. Hand length (HnL) 7.4 mm its length 31.0% of SVL. Palmar tubercle bifid, about two-thirds the length of oval thenar tubercle (Fig. 7). Supernumerary palmar tubercles present, rounded to elongated, and slightly elevated; subarticular tubercles large, round, and conical; fingers without lateral fringes; disks on all fingers rounded apically and extensively expanded (Fig. 7); disk of finger III equal in diameter to the tympanic annulus; disks bearing ventral pads; finger I shorter than II when appressed (Fig. 7). Relative lengths of appressed fingers III>IV>II>I. Subarticular tubercles 1–1–2–2.

Hindlimbs slender; foot length (FtL) 12.1 mm, 50.8% of SVL. Toe webbing and toe fringes absent. Relative lengths of appressed toes IV>V>III>II>I. Discs of the toes expanded; width of adjacent phalange 53.7% of disc of toe IV; disc of toe III does not reach penultimate subarticular tubercle of toe IV; toe V beyond that of the level of penultimate subarticular tubercle of toe IV. Femur length (FL) 13.6 mm, tibia length (TL) 14.5 mm, its length is equivalent to 60.9% of SVL. Subarticular tubercles 1–1–2–3–2; supernumerary plantar tubercles numerous, suboval, and low; inner metatarsal tubercle oval; outer metatarsal tubercle rounded, prominent, and smaller than inner metatarsal tubercle. Diameter outer metatarsal tubercle 52.8% of inner metatarsal tubercle; outer tarsal fold absent; inner tarsal fold short. Numerous supernumerary plantar tubercles rounded and barely visible; subarticular tubercles large, round, and conical; toes without lateral fringes; no webbing. Cloacal sheath absent; subcloacal tubercles absent.

***Color of holotype in preservative*** (Fig. 4). Dorsum and flanks dark brown; hands in dorsal view, with fingers I and II cream-colored, while fingers III and IV brown with cream-colored bars; dorsal surfaces of the thigh with diffuse dark-brown transversal bars; hidden surfaces of thighs pale brown; venter light brown with a dark-brown suffusion and mottled brown; ventral surfaces of hindlimbs and forelimbs dark brown with a cream-colored suffusion; edge of chin with irregular blotches of dark brown; hands, in ventral view, with palmar tubercle cream-colored and palmar region dark brown.

***Color of holotype in life*** (Fig. 5). Dorsal surfaces of body and limbs pink-orange; flanks salmon and sides of the head pink-orange; venter reddish cream-colored on chest and belly, cream-colored on throat; axillary region, groin, and anterior thigh pale orange; ventral surfaces of thighs light brown; iris gray, medially reddish, with black reticulations.

##### Variation of type series

(Fig. 5, Table 4). In this section, coloration refers to specimens in life and is based on field notes and digital photographs, unless otherwise noted. Dorsal coloration reddish brown with mottled, dark-brown chevrons, usually surrounded by a thin band of lighter color; canthal stripe black; dorsal surfaces of thigh with dark-brown transversal bars; axillary region, groin, and anterior thigh bright orange (e.g., IAvH-Am-10283, IAvH-Am-10276; Fig. 5) or uniformly dark brown (e.g., IAvH-Am-10267–68, IAvH-Am-10272; Fig. 5). An adult female (IAvH-Am-10277) has a gold paravertebral line (Fig. 5). Labial bars dark brown, and postorbital and supratympanic stripe dark (e.g., IAvH-Am-10268, IAvH-Am-10270, IAvH-Am-10272, IAvH-Am-10276–7; Fig. 5). In IAvH-Am-10270 and IAvH-Am-10276, flanks with oblique, irregular, dark-brown bars (Fig. 5); IAvH-Am-10267 with a W-shaped, light-brown marking on scapula; some specimens with a dark-brown interorbital bar (e.g., IAvH-Am-10268, IAvH-Am-10273–4, IAvH-Am-10279–10280). *Pristimantis chamezensis* is metachromatic, being lighter in color at night. Teeth positioned posterior to level of choanae and widely separated from each other, which vary between eight to nine. The variation in the skin texture is noteworthy (Fig. 5), varying from smooth (e.g., IAvH-Am-10283) to shagreen with scattered tubercles (e.g., IAvH-Am-10267, IAvH-Am-10277). The SVL of adult males ranges from 19.6 to 23.7 mm (Table 4), and the SVL of adult females ranges from 19.0 to 24.9 mm (Table 4). The HW 35.9–40.3% of SVL in adult males and 41.2–44.1% in adult females. ED 61.6–75.0% of ETS in adult males and 59.1–74.9% in adult females. UEW 58.0–77.6% of interorbital distance (IOD) in adult males and 54.6–78.2% in adult females. TD 39.1–56.0% of ED in adult males and 33–41.8% in adult females. HnL in adult males 29.3% of SVL and 31.2% in adult females. FtL in adult males 45.6–50.4% of SVL and 44.9–51.8% in adult females.

##### Distribution and natural history.

This species is only known from the type locality at an altitude between 2125–2160 m a.s.l. in an Andean and relictual cloud forest in the Casanare region on the eastern flank of the Cordillera Oriental of Colombia (Fig. 1). The locality is within the Cordillera Oriental montane forest ecoregion (*sensu*Dinerstein et al. 1995) in the Andean region (Middle Orobiome). The forest (Fig. 2) is unaffected by human activities and is typified by a canopt of medium-height (up to 20 m). The annual precipitation is between 4600 and 5600 mm with bimodal seasonality. Specimens were found active during the second annual rainy season (August to November) at a temperature of 14 °C resting on mosses and lower leaves of shrubs and ferns in the undergrowth. *Pristimantis chamezensis* is syntopic with an undescribed species of genus *Pristimantis*.

##### Etymology.

The specific epithet is derived from the Municipality of Chámeza, a geopolitical area where the type locality is located. We decided on this name using a citizen science approach, where expert scientists and local people met and discussed a list of possible names and their corresponding meanings. There was consensus on *P. chamezensis* as the preferred name.

##### Conservation status.

The direct evaluation of the landscape units (e.g., broad-leaved forest) at the type locality, as well as the map of land cover of Colombia (CORINE Land Cover, IDEAM 2010), allowed us to identify a rapid reduction and low connectivity of its habitat. Based on land cover maps of Chameza’s forest, the potential extent of occurrence is 301,624 km^2^. Consequently, we propose to categorize *P. chamezensis* as Vulnerable using the criteria B2a (IUCN Red List Categories and Criteria 2019).

## Discussion

### Colombian diversity of the genus *Pristimantis* in a biogeographical context

**Figure 8.**
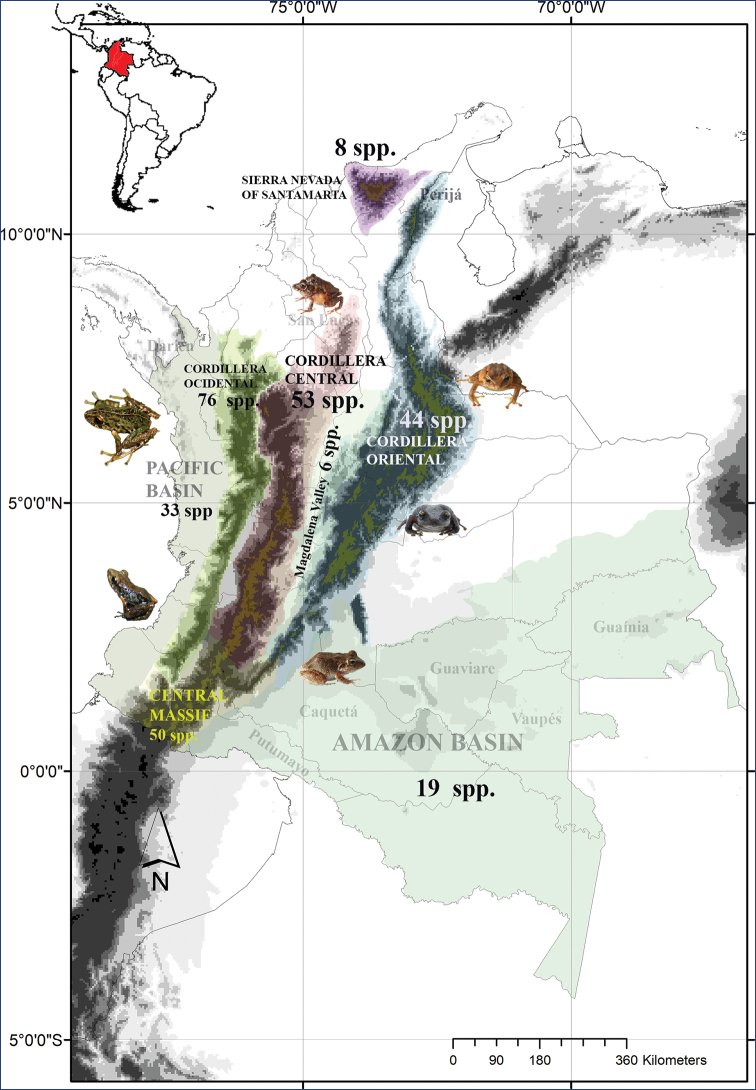
Geographic diversity of frogs of the genus *Pristimantis* in Colombia; the numerical values correspond to the number of species reported in each region.

The genus *Pristimantis*, with 556 described species, comprises of a substantial number of identified taxa (Frost 2020). Colombia harbors 40% of this diversity. The Andean Cordilleras harbor 183 species (Acosta-Galvis 2020), evidencing the high rate of speciation and endemism of the genus in this ecoregion (Lynch 1999), while in the lowlands (Pacific, Middle Magdalena, and Amazon basins) there are just 52 species. The diversity of *Pristimantis* of the Andean-Cordillera and Sierra Nevada of Santa Marta reflects the geological history of these mountains (Lynch and Ruiz-Carranza 1985; Lynch et al. 1997). Consequently, the geological formations of the Cordillera occidental (25 Ma old, with the greater species richness), Cordillera Central, and the Central Massif exhibit a 30% similarity of species. While, the Cordillera Oriental (10 Ma old; Gregory-Wodzicki 2000) and Sierra Nevada de Santa Marta (2.6 Ma old; Idárraga et al. 2011) have allowed the evolution of an unparalleled diversity with a high degree of endemism (Lynch et al. 1997) (Fig. 8).

Despite this rough correspondence between the geological history of the Colombian Andes and *Pristimantis* diversity, the inventory of species in each region is far from being completed. Socio-political factors affecting the various regions of Colombia have limited scientific access, leaving several crucial regions with pronounced gaps in our knowledge of amphibians. Among these regions, we highlight the northern lowland regions of the upper Amazon, including Putumayo, Caquetá, Guaviare, Guainía, and Vaupés departments, as well as neighboring areas such as the Darien region. Additionally, some other unsampled areas are the tropical rainforests in the Pacific basin and the Andean region, such as the Serranias of Perijá and San Lucas, southern Cordillera Oriental (including the Andean-Amazonian foothills) and mountainous areas associated with the Orinoco drainage (Fig. 8).

Over the past six years of scientific studies in unexplored mountainous areas within the Orinoco drainage, including cloud forests and foothills of the Cordillera Oriental, several species of *Pristimantis* have been described (e.g., Acosta-Galvis et al. 2010; Acosta-Galvis and Alfaro-Bejarano 2011; Pedroza-Banda et al. 2014; Rivera-Correa et al. 2016; Acevedo et al. 2020; Ospina-Sarria and Angarita-Sierra 2020). However, there is still a long way to go to characterize the amphibian fauna of this region.

### Phylogenetic relationships of *Pristimantis chamezensis*

In our research, the integration of morphological and genetic data allowed us to establish that *P. chamezensis* is distinct from the other 13 *Pristimantis* species from Andean and sub-Andean forests on the eastern flank of the Cordillera Oriental. Taking into account the agreement between all phylogenetic analyses revealing a supported monophyletic group comprised of *P. chamezensis*, *P. carranguerorum*, *P. bowara*, *P. lutitus*, *P. medemi*, *P. nicefori*, and *P. savagei*, as well as the altitudinal (450–4170 m a.s.l.) and longitudinal distribution of those species along the Andean and sub-Andean forest on the eastern flank of the Cordillera Oriental (almost all are syntopic except by *P. lutitus* and *P. nicefori* from the western flank), it is probable that the origin of the new species and the radiation of the monophyletic group may have occurred at higher altitudes within this region. It might be possible that these *Pristimantis* lineages show the same pattern of recent diversification due to climatic changes, as seen in both, a high altitude dendrobatid frog (*Hyloxalus felixcoperari* Acosta-Galvis & Vargas-Ramírez, 2018) and a group of Andean anoles (*Anolis heterodermus* species group; Vargas-Ramírez and Moreno-Arias 2014) from the middle part of the eastern Cordillera.

Nevertheless, the generalized low support of the phylogenies emphasizes the need to increase the molecular dataset to reveal with confidence the evolutionary relationships within *Pristimantis*. This is clear from the recent changes in the phylogenetic position of several species (e.g., Hedges et al. 2008; Padial et al. 2014; Reyes-Puig et al. 2020). In addition, it is still required to incorporate a large number of unassigned Colombian taxa into evolutionary based species groups. There are about 117 species not yet analyzed using phylogenetic methods.

Our phylogenetic analyses unequivocally revealed that *P. chamezensis* is part of the subgenus Pristimantis. However, we do not force its allocation into one of the several species group (Hedges et al. 2008; Padial et al. 2014; Acevedo et al. 2020). Although our results validate some arrangements (e.g., *conspicillatus* or *danae* species groups; Fig. 3), some other individual assignments are weakly supported, and do not correspond to arrangements within the already proposed groups. Among the examples that we can identify, is the nesting of *P. chamezensis* with *P. nicefori*, which was formerly assigned within the *unistrigatus* group by Hedges et al. (2008) and later transferred to unassigned species group by Padial et al. (2014). Likewise, the close relationship of the *chamezensis*+ *P. nicefori* clade with the *P. lutitus* + *P. medemi* + *P. carranguerorum* clade (Fig. 3) is inconsistent with previous species groups assignments; *P. medemi* and *P. carranguerorum* were assigned to the *conspicillatus* species group by Hedges et al. (2008) and, later, validated by Padial et al. (2014). Additionally *P. lutitus* (Fig. 3), which was formerly assigned to the *unistrigatus* species group but subsequently transferred to an unassigned species group by Padial et al. (2014) and later inferred as sister to *P. anolirex* by Rivera et al. (2016).

## Conclusion

*Pristimantis chamezensis* is described as an endemic species from Chámeza forest. This new species is closely related to *P. carranguerorum*, *P. bowara*, *P. lutitus*, *P. medemi*, *P. nicefori*, and *P. savagei*.

## Supplementary Material

XML Treatment for Pristimantis chamezensis

## Supplementary materials

Supplementary material 1 Additional specimens examined. IAvH-Am: Alexander von Humboldt Biological Resources Research Institute, Villa de Leyva, Colombia; MPUJ: Lorenzo Uribe Museum of Natural History, Pontificia Universidad Javeriana, Bogotá D.C., Colombia Data type Additional Specimens of genus *Pristimantis* of Cordillera Orienta of Colombial https://binary.pensoft.net/file/443389This dataset is made available under the Open Database License (http://opendatacommons.org/licenses/odbl/1.0/). The Open Database License (ODbL) is a license agreement intended to allow users to freely share, modify, and use this Dataset while maintaining this same freedom for others, provided that the original source and author(s) are credited.Andrés R. Acosta-Galvis, Ana M. Saldarriaga-Gomez, Beatriz Ramirez, MarioVargas-Ramirez

Supplementary material 2 Figure S1 Data type image Explanation note Maximum likelihood inference tree showing the evolutionary relationships of Pristimantis chamezensis sp. nov. (bold) and its 58 most closely related Pristimantis species based on 528 bp of the 16S rRNA gene. Numbers before nodes: thorough maximum likelihood (ML) bootstrap percentages left and Bayesian analysis (BA) posterior probability values right. Bootstrap values below 50% and posterior probabilities below 0.5 not shown. Outgroup taxon removed. https://binary.pensoft.net/file/443390This dataset is made available under the Open Database License (http://opendatacommons.org/licenses/odbl/1.0/). The Open Database License (ODbL) is a license agreement intended to allow users to freely share, modify, and use this Dataset while maintaining this same freedom for others, provided that the original source and author(s) are credited.Andrés R. Acosta-Galvis, Ana M. Saldarriaga-Gómez, Beatriz Ramírez, Mario Vargas-Ramírez

Supplementary material 3 Figure S2 Data type image Explanation note Bayesian (BA) and maximum likelihood (ML) topologies (right and left respectively) obtained based on 1185 bp of the 16S rRNA + COI genes. Numbers before nodes: BA posterior probability values and ML bootstrap percentages. Bootstrap values below 50% and posterior probabilities below 0.5 not shown. https://binary.pensoft.net/file/443391This dataset is made available under the Open Database License (http://opendatacommons.org/licenses/odbl/1.0/). The Open Database License (ODbL) is a license agreement intended to allow users to freely share, modify, and use this Dataset while maintaining this same freedom for others, provided that the original source and author(s) are credited.Andrés R. Acosta-Galvis, Ana M. Saldarriaga-Gómez, Beatriz Ramírez, Mario Vargas-Ramírez
