# Supplementary material for: A new Terrarana frog of genus Pristimantis from an unexplored cloud forest from the eastern Andes, Colombia
Source: Zookeys. 2020 Aug 19;961:129–56. doi: 10.3897/zookeys.961.51971 (PMC7449990; doi:10.3897/zookeys.961.51971)
Supplement: Supplementary material 1 — Additional specimens examined. IAvH-Am: Alexander von Humboldt Biological Resources Research Institute, Villa de Leyva, Colombia; MPUJ: Lorenzo Uribe Museum of Natural History, Pontificia Universidad Javeriana, Bogotá D.C., Colombia [file zookeys-961-129-s001.docx]

**Supplementary material 1.** Additional specimens examined. IAvH-Am: Alexander von Humboldt Biological Resources Research Institute, Villa de Leyva-Colombia; MPUJ: Lorenzo Uribe Museum of Natural History, Pontificia Universidad Javeriana, Bogotá, Colombia.

***Pristimantis anolirex***. COLOMBIA: Boyacá Department, Rondón Municipality, Vereda Juan Vásquez, 5°24'34.1''N;73°13'16.8''W, 3448 m a.s.l., IAvH-Am-13138. Norte de Santander Department, Arboledas Municipality, Corregimiento Castro, Vereda Quebrada Grande, Los Cristales and Llano Grande farms, 7°40'15.5"N;72°57'59.2"W, 3570 m a.s.l., IAvH-Am-8620–8634; Cachira Municipality, Vereda Santo Domingo, Siete Lagunas farm, 3665 m a.s.l., 7°40'15.5"N;72°57'59.2"W, IAvH-Am-8609–8619; Vereda Quebrada Grande, site Torres Mague, Tamá National Natural Park, IAvH-Am-8719. Santander Department, Santa Barbará Municipality, Vereda Volcanes, Páramo of Santurban, 7°4'22.91"N;72°51'39.11"W, IAvH-Am-15599–15600; Vereda Los Tutos, Alto de las Cruces, 7° 4'23.01"N;72°51'39.12"W, IAvH-Am-15601–611; Vereda La Salina, Hacienda San Francisco, 7°00'23.9"N;72°51'41.5"W, 3324-3478 m a.s.l., IAvH-Am-15645–15663.

***Pristimantis carranguerorum***. COLOMBIA: Boyacá Department, Pajarito Municipality, Police inspection Corinto, 1700 m a.s.l., IAvH-Am-2329–50, IAvH-Am-6475; middle basin of the river Cusiana, The Guayabal farm, 1300 m a.s.l., IAvH-Am-6626, IAvH-Am-9192–9; Garagoa Municipality, road between Garagoa to Juntas, 1380 m a.s.l., IAvH-Am-7685–9; Campohermoso Municipality, vereda Huerta Vieja, 1300 m a.s.l., IAvH-Am-7690–2; Páez Municipality, Vereda Mochilero, 1300 m a.s.l., IAvH-Am-7693–9. Casanare Department, Chámeza Municipality, Vereda Centro Norte, Chámeza forest, eastern flank of the Cordillera Oriental, 05°15’24.4”N;72°53’51.6”W, 2140 m a.s.l., MUJ 7073, MUJ 7084.

***Pristimantis frater*.** COLOMBIA: Boyacá Department, Pajarito Municipality, middle basin Cusiana River, El Guayabal farm, 4°40'0"N;75°46'39"W, IAvH-Am-6880–1. Casanare Department, Yopal Municipality, Corregimiento El Charte, Vereda Rincón del Soldado, Aqueduct veredal El Infierno creek, 877 m a.s.l., 5°23'17.7"N;72°29'8.9"W, IAvH-Am-10801–3, IAvH-Am-11951; Forest Aqueduct, 5°23'48.8"N;72°29'14.6"W, IAvH-Am-10804–5; Tauramena Municipality, Vereda Chaparral, Forest Pozo Buenos Aires (BAX-30), behind of Equion Camp, 4°59'31.1"N;72°43'1.8"W, 372 m a.s.l., IAvH-Am-11251–6, IAvH-Am-11258–11260. Meta Department, Vista Hermosa Municipality, Caño Sardinata, Sierra de La Macarena Natural National Park, IAvH-Am-3394; Duda River, Primatologic station Puerto Chamuza, Tinigua Natural National Park, IAvH-Am-5324, IAvH-Am-5327–8; Villavicencio Municipality, Vereda Buenavista, Caño Carrilo, 1004 m a.s.l., 4°8'22"N;73°40'50"W, IAvH-Am-8502.

***Pristimantis lynchi****.* COLOMBIA: Boyacá Department, Arcabuco Municipality, oak forests, 5°46'4.04''N;73°25'18.44''W, 2805 m a.s.l., IAvH-Am-15764; Aquitania Municipality, Páramo of Toquilla, Vadohondo site, 2660 m a.s.l., IAvH-Am-4696 (ex. IND-AN-4696), IAvH-Am-4695 (ex. IND-AN 4695), IAvH-Am-4694 (ex. IND-AN 4694), IAvH-Am-4693 (ex. IND-AN 4693); Páramo of Franco, vereda Toquilla, 5°30'35.7''N;72°41'52.2''W, 3349 m a.s.l., IAvH-Am-13158; Cerinza Municipality, Vereda Chital, Las Animas creek, 5º 58' 20.64"N;72º 59' 21.73"W, 3258 m a.s.l., IAvH-Am-15817;Vereda Toba, 5º 57' 15.9"N;72º 59' 21.08"W, 3328 m a.s.l., IAvH-Am-15807–8; Los Colorados Creek, 6° 3'52.38"N;72°54'17.92"W, 3584 m a.s.l., IAvH-Am-15809–15816; Cómbita Municipality, Vereda Santa Bárbara, La Empedrada Lake, Páramo of El Valle, 5°44'52.4"N;73°22'33.6"W, 3551 m a.s.l., IAvH-Am-11007–17, IAvH-Am-15770, 5°44'56.436'''N;73°21'6.6234''W, 3242 m a.s.l., IAvH-Am-15755, IAvH-Am-15757–15760; Mongua Municipality, Vereda Sirguazá, 5°44'49.1''N;72°40'15.24''W, 2386 m a.s.l., IAvH-Am-15860; Monguí Municipality, Vereda Pericos, Sector El Santuario, 5°44'9.3''N;72°49'35.8''W, 3333 m a.s.l., IAvH-Am-15854–9; Susacón Municipality, Vereda Desaguadero, Páramos of Guantiva-La Rusia complex, 6°12'47.3''N;72°46'58.5''W, 3455 m a.s.l., IAvH-Am-13166–13172; Tutazá Municipality, Vereda Tobal, Los Colorados Creek, 6º 3' 52.8"N;72º 54' 18.4"W, 3542 m a.s.l., IAvH-Am-15818–19, IAvH-Am-15822, 6º3'44.82"N;72º54'10.69"W, 3503 m a.s.l., IAvH-Am-15820–1, IAvH-Am-15823.

***Pristimantis medemi.*** COLOMBIA: Casanare Department, Chámeza Municipality, Vereda Centro Norte, Chámeza forest, eastern flank of Cordillera Oriental, 1760–2140 m a.s.l., 05°15’24.4”N;72°53’51.6”W, MUJ 7054, MUJ 7056–59, MUJ 7061–7071, MUJ 7095–98;Yopal Municipality, Corregimiento Charte, vereda Rincón del Soldado, La Primavera farm, forests head La Aguazula creek, 05°23´00”N;72°29´55.6”W, 1003 m a.s.l., IAvH-Am-11960–6; La Primavera forests 05°22´46.5”N;72°30´2.8”O, 959 m a.s.l., adult males , IAvH-Am-11967–11970; El Secreto I forest, 05°23´26.8”N;72°29´21.9”W, 1015 m a.s.l., IAvH-Am-11971–3; El Infierno creek, 05°23´17.7”N;72°29´8.9”W, 877 m a.s.l., IAvH-Am-11956–7, IAvH-Am-11958–9; Corregimiento El Charte, border veredas La Primavera-Rincón del Soldado, 05°23´15.4”N;72°28´42.4”W, 860 m a.s.l., IAvH-Am-11954–5; Corregimiento El Morro, head Cravo Sur river, 05°26´59.5”N;72°27´22.1”W, 610 m a.s.l., IAvH-Am-11952; Vereda El Aracal, 05°30´32.6”N;72°24´36.2”W, 1324 m a.s.l., IAvH-Am-11974–5; Cauteña creek, 05°30´15.4”N;72°24´17.2”W, 839 m a.s.l., IAvH-Am-11976; Vados creek, 05°29´50.6”N;72°24´22.2”W, 831 m a.s.l., IAvH-Am-11977–81. Meta Department, Villavicencio Municipality, Vereda El Carmen, Caño Buque, Forestal Reserve Quebrada Honda, 921-1021 m a.s.l., IAvH-Am-8453–8469; 4°11'26.6"N;73°36'45.9"W, 1021 m a.s.l., IAvH-Am-8530; Vereda Buenavista, Caño Carrillo,4°8'22.9"N;73°40'50"W, 1004 m a.s.l., IAvH-Am-8470–73,IAvH-Am-8475–8481; Vereda Vanguardia Alta, Forestal Reserve Vanguardia, road Biopark Los Ocarros, 4°9'42.2"N;73°41'15.6"W, 456 m a.s.l., IAvH-Am-8482–8495.

***Pristimantis savagei*.** COLOMBIA: Casanare Department, Yopal Municipality, Corregimiento El Charte, vereda Rincón del Soldado, veredal aqueduct of El Infierno creek, 05°23´17.7”N;72°29´8.9”O, 877 m a.s.l., IAvH-Am-10782; El Secreto I forest, 05°23´26.8”N;72°29´21.9”O, 1015 m a.s.l., IAvH-Am-10795–800; Corregimiento El Morro, Vereda Aracal, El Aracal Forests, 5°30´32.6”N;72°29´36.2”,1324 m a.s.l., IAvH-Am-10783–87, IAvH-Am-10790–2; Cauteña creek, 5°30´15.4”N; 72°24´17.2”,839 m a.s.l., IAvH-Am-10788–9; Vereda El Progreso, Forest of Heads, 5°30´27.9.4”N;72°24´17.2”,1811 m a.s.l., IAvH-Am-10793–4. Cundinamarca Department, Guayabetal Municipality, Vereda Portachuelo (Manzanares) 1900 m a.s.l., IAvH-Am-5028–9, IAvH-Am-7119–7126; close to Guayabetal, IAvH-Am-7985, IAvH-Am-7987; Medina Municipality, IAvH-Am-7971–3. Meta, Department, Villavicencio Municipality, Vereda Buena Vista, Caño Carillo, 4°9'42.2"N;73°41'15.6"W, 1004 m a.s.l., IAvH-Am-8474, IAvH-Am-8510; La U, 4°8'34.9"N;73°40'46.3"W, 1155 m a.s.l., IAvH-Am-8501, IAvH-Am-8506-9; Vereda El Carmen, Caño Buque, 1021 m a.s.l., IAvH-Am-8496–8500, IAvH-Am-8504–5; Forestal Reserve Quebrada Honda, 4°8'22"N;73°40'50"W, 921 m a.s.l., IAvH-Am-8503.

***Pristimantis vilarsi*.** COLOMBIA: Amazonas Department, Leticia Municipality, Santa Sofia Island, IAvH-Am-3403. Caquetá Department, Mesay River, Puerto Abeja station, Chibiriquete National Natural Park, 0°4'27"N;72°27'5"W, 240 m a.s.l. , IAvH-Am-6544–5. Guainía Department, Caño Rico, near Cerro Mina, IAvH-Am-6188. Meta Department, mouth Caño Cabra, Sierra de La Macarena National Natural Park, IAvH-Am-2386–9; Vista Hermosa Municipality, Caño Sardinata, Sierra de La Macarena National Natural Park, 1300 m a.s.l., IAvH-Am-3389–91, IAvH-Am-3393; Duda River, Puerto Chamuza research station, Tinigua National Natural Park, IAvH-Am-5317–9, IAvH-Am-5321, IAvH-Am-5325. Vaupés Department, Mitú Municipality, La Urania, San Antonio cave, IAvH-Am-271–3, IAvH-Am-459, IAvH-Am-463, IAvH-Am-3400–1; Raudal of Yuruparí river, IAvH-Am-595; Taraira Municipality, Taraira Lake, Caparú Biological station, IAvH-Am-6997–9. Vichada Department, Cumaribo Municipality, Main cab, administrative center, El Tuparro National Natural Park, IAvH-Am-310–1, IAvH-Am-2305; Corregimiento of Santa Rita, Tomo River, El Tuparro National Natural Park,1°39'54"N;76°8'28"W, 240 m a.s.l., IAvH-Am-7493–7, IAvH-Am-7510, IAvH-Am-9272–3.

***Pristimantis bogotensis*.** COLOMBIA: Cundinamarca Department, Bogotá, D.C., road km 9, Bogotá-Choachi, 3000 m a.s.l., IAvH-Am-59; km 11 road Bogotá-Choachí, 3000-3200 m a.s.l., IAvH-Am-8747–1, IAvH-Am-8753–8788; páramo outside eastern of Bogotá, IAvH-Am-506–8, IAvH-Am-546, IAvH-Am-549, IAvH-Am-552–3, AvH-Am-9109–23; páramo Cruz Verde, IAvH-Am-3010–3032; Sede Venado del Oro, older INDERENA, IAvH-Am-665, IAvH-Am-2606, IAvH-Am-2613, IAvH-Am-2617, IAvH-Am-3545, IAvH-Am-7956–9; Los Tanques de Vitelma, La Marranera farm, km 6 road to El Delirio farm, IAvH-Am-3550–65; Guadalupe hillside, IAvH-Am-3721–26; páramo of Monserrate, El Granizo farm, 3100 m a.s.l., IAvH-Am-3838; mountains of Monserrate, 0°4’27”N;72°27’5”W, 3100 m a.s.l., IAvH-Am-6555; páramo of Sumapaz, high valley of Curubital river, 3800 m a.s.l., IAvH-Am-3856–7, IAvHAm-3859–61, IAvH-Am-3863, IAvH-Am-3865–74, IAvH-Am-3876–7, IAvH-Am-3879–3881; Fomeque Municipality, Chingaza National Natural Park, IAvH-Am-2374–7, IAvH-Am-7171–2, IAvH-Am-7364–76, IAvH-Am-7378–80, IAvH-Am-9224–9271; Chingaza National Natural Park, Chuza sector, 3300 m a.s.l., IAvH-Am-5408–5416; Junín Municipality, Chingaza National Natural Park, border Carpanta Reserve, 3000 m a.s.l., IAvH-Am-5345–6; Chingaza National Natural Park, La Playa sector, 4°32’22”N;73°45’42.7”W, IAvH-Am-9284–5; La Calera Municipality, Paramo of Palacios km 22 road to Cementos Samper, 3200-3500 m a.s.l., IAvH-Am-3115–44, IAvH-Am-3500–2; right margin road, reten Los Patios, IAvH-Am- 9802–6. Meta Department, San Juanito Municipality, Chingaza National Natural Park, San José, Planes de San Luis, road to San Juanito, 4°30’30” N ;73°41’23” W, 2966 m a.s.l., IAvH-Am-9297.
