# Supplementary figures and images for: A new Terrarana frog of genus Pristimantis from an unexplored cloud forest from the eastern Andes, Colombia
Source: Zookeys. 2020 Aug 19;961:129–56. doi: 10.3897/zookeys.961.51971 (PMC7449990; doi:10.3897/zookeys.961.51971)

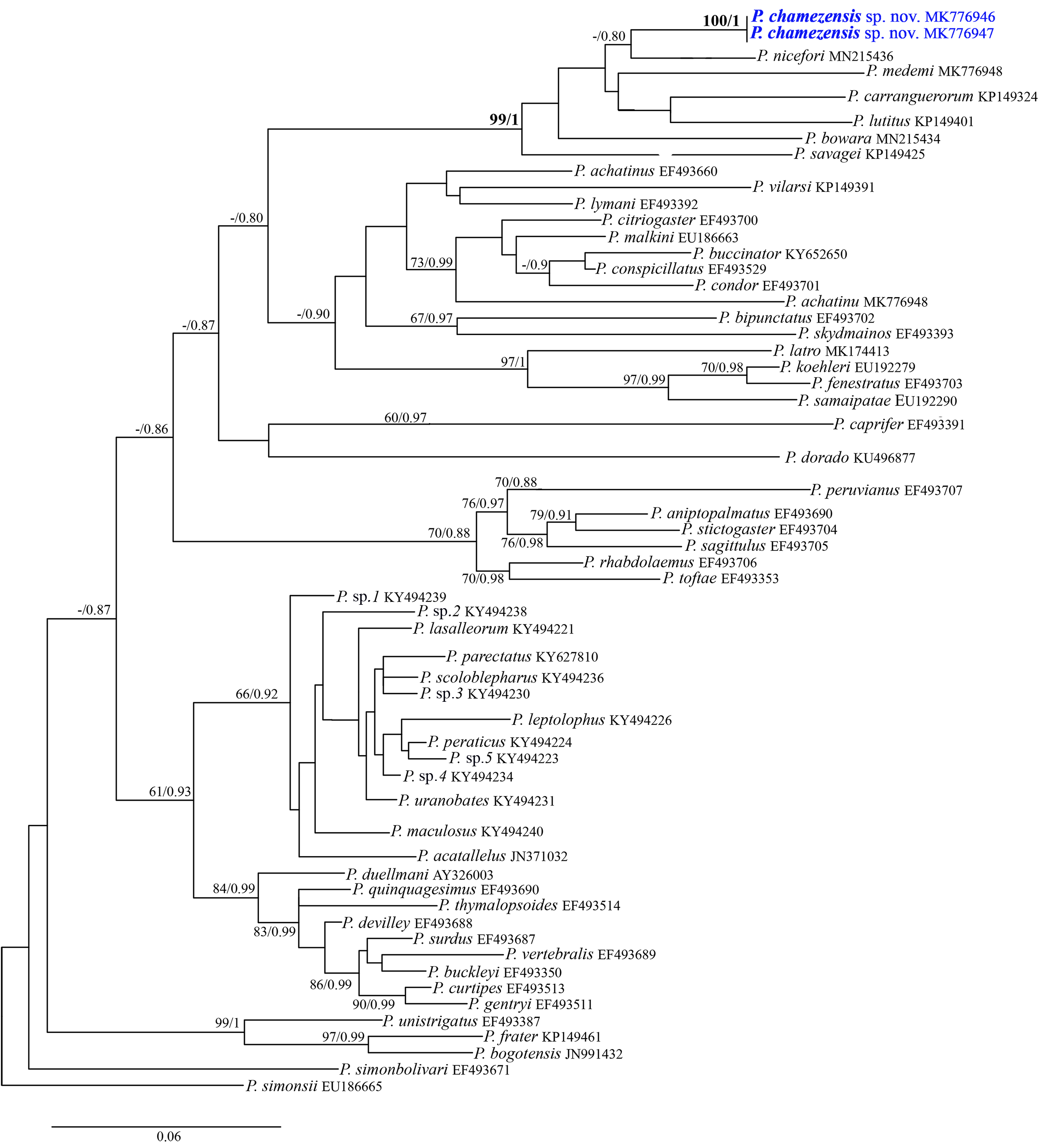

Supplement: Supplementary material 2 — Figure S1 [file zookeys-961-129-s002.tif]

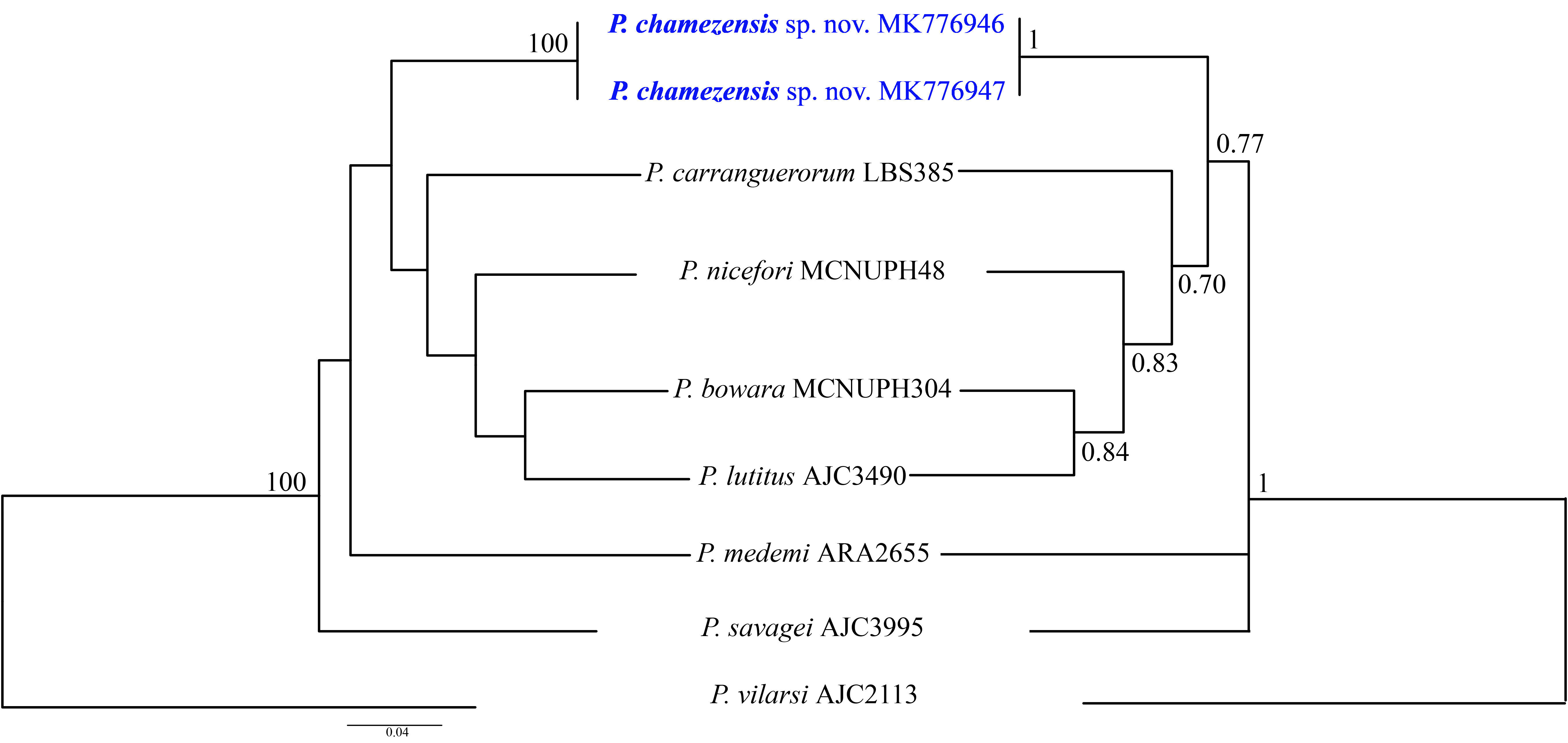

Supplement: Supplementary material 3 — Figure S2 [file zookeys-961-129-s003.tif]
